# Knowledge and perception about dental implants among undergraduate dental students

**DOI:** 10.1038/s41405-018-0009-1

**Published:** 2019-03-14

**Authors:** Arati Sharma, Bijay Kumar Chaudhari, Bidhan Shrestha, Pramita Suwal, Prakash Kumar Parajuli, R. K. Singh, Surya Raj Niraula

**Affiliations:** 1 Department of Prosthodontics, CODS, B.P. Koirala Institute of Health Sciences, Dharan, Nepal; 2 Department of ENT, NAMS, Bir Hospital, Kathmandu, Nepal; 3 Department of Prosthodontics, Kantipur Dental College and Hospital, Kathmandu, Nepal; 4 Department of Public Health and Community Medicine, B.P. Koirala Institute of Health Sciences, Dharan, Nepal

## Abstract

**Introduction:**

Awareness about dental implants is increasing among dental patients, which demands a higher level of competence for dental students. So, the objective of this study was to assess the knowledge and perception of undergraduate dental students about dental implants.

**Materials and methods:**

This cross-sectional questionnaire-based survey was conducted after taking ethical clearance and approval from the Institutional Review Committee of B.P. Koirala Institute of Health Sciences and informed consent from each dental college of Nepal. The sample included all those students who were present at the time of survey. Data collection were carried out during the academic schedules of the colleges, supervised, and monitored by the investigators themselves. Collected data were coded, entered in Microsoft excel 2013 and descriptive analysis was carried out.

**Results:**

A majority of the total (54.6%) and 59.2% of 5^th^ year respondents perceived to be moderately well-informed about dental implants. The main advantage of dental implants was thought to be longevity by 53.1% of total and 48.4% of 5^th^ year students; only 27.6% of the total and 42.2% of 5^th^ year students said the main advantage of dental implants is they are more conservative than other tooth-replacement modalities. Highest percentage of the total respondents (31.9%) said most important factor for implant success to be implant type and material, whereas 59.8% of 5^th^ year students said case selection. Those who felt dental implants require additional oral hygiene maintenance and care by the patient and dentist were 58.4% of total and 75.1% of 5^th^ year students. Over two-thirds (67.5%) of total thought that economic feasibility will limit use of dental implants in Nepal. The difficulty encountered to place implants was perceived to be average by 56.8% of total and 58.1% of 5^th^ year. There were differences in the perception and knowledge at different academic levels, but not as expected.

**Conclusions:**

It could not be concluded that knowledge about dental implants increased with increase in academic level. Even at the late-clinical year a majority of students gave unsatisfactory responses.

## Introduction

Implant dentistry has evolved into the mainstream of restorative practices all over the world.^1^ It has mainly two phases; a surgical phase and a prosthodontic phase. For centuries, there were ways to replace the crown but not the root but root replacement is now possible.^2^ Endoseous dental implants are alternative tooth roots and implant-supported prostheses are considered the best substitute for missing teeth.

Awareness about dental implants is increasing among the general public and more and more patients are seeking information about dental implants.^3–9^ It is therefore useful to gauge the level of information about dental implants among dental students. All undergraduate dental students require basic knowledge about dental implant therapy so that they can educate and guide patients to undergo implant therapy whenever appropriate.

America, Australia, and many European countries have included aspects of implant dentistry in the curriculum for undergraduate dental students, conducted curriculum surveys, held consensus workshops regarding the concern and modified their curricula accordingly.^10–13^ In Asia, particularly in developing countries like Nepal, there is little evidence in the literature regarding this aspect. A survey of 92 dental schools found that only 49% of dental schools offered surgical and prosthodontics courses related to implants in which students mainly observe and of these only 29% of dental schools were from Asia.^14^ The percentage of hands-on courses on implants for undergraduates is higher in North America and Europe than in Asia, South America, and Africa.^14^

Bachelor of Dental Surgery (BDS) is equivalent to Doctor of Dental Surgery (DDS). BDS is four and a half years plus one year compulsory internship program in Nepal. The first 2 years are preclinical and from 3^rd^ year clinical posting starts. This study was conducted to ascertain the level of information about dental implants among undergraduate dental students from 1^st^ year to 5^th^ year and thus, to know whether there is a need to survey the curriculum and teaching materials and methods.

## Materials and methods

This cross-sectional questionnaire-based survey was conducted from June 2016 to June 2017 after taking ethical clearance and approval from the research committee of the B.P. Koirala Institute of Health Sciences and informed consent from each dental college of Nepal. There were 11 dental collages with students at all academic levels. Enquiry was made about the number of students in different academic years of each college and the total number of students was thus calculated. The study population was all undergraduate dental students of Nepal. The sample included all those students who were present at the time of survey and excluded those who were absent. Data were collected through a pre-used questionnaire taken from previous study.^15^ Minor modifications relevant to the Nepalese population was made in the questionnaire after a pilot study. Data collection were carried out during term-time supervised and monitored by the investigators themselves. Collected data were coded, entered in Microsoft excel 2013 and descriptive analysis carried out.

## Results

The total number of students was 2400. The number of students present (number of questionnaires distributed) was around 1810, out of which 1700 responded completely. Thus, the true response rate was 93.9%. Some aspects of demographic variables like age, sex, academic levels, and some other aspects of the survey have been published previously.^16^ Distribution of students according to sex, age, and response rate have been shown in the tables below (Tables 1–3). Responses to the questions at each academic level have been presented in Tables 4, 5.

**Table 1:** Distribution of students on the basis of sex at the academic years

Academic year	Male *n* (%)	Female *n* (%)
1^st^ year	88 (18.4)	288 (23.6)
2^nd^ year	85 (17.7)	231 (19.0)
3^rd^ year	91 (19.0)	239 (19.6)
4^th^ year	108 (22.5)	229 (18.7)
5^th^ year	107 (22.3)	234 (19.1)
Total	479 (100.0)	1221 (100.0)

**Table 2:** Distribution of students on the basis of age at the academic years

Academic year	Age (Mean ± SD = 21.47 ± 2.07)	
	≤21 years *n* (%)	>21 years *n* (%)
1^st^ year	376 (48.6)	0 (0.0)
2^nd^ year	312 (40.4)	4 (0.4)
3^rd^ year	82 (10.6)	248 (26.7)
4^th^ year	3 (0.4)	334 (36.0)
5^th^ year	0 (0.0)	341 (36.8)
Total	773 (100.0)	927 (100.0)

**Table 3:** Response rate at each academic year

Academic year	Estimated number of students (*n*)	Students present (*n*)	Students responded completely (*n*)	Complete response rate (%)
1^st^ year	540	399	376	94.2
2^nd^ year	540	382	316	82.7
3^rd^ year	460	342	330	96.5
4^th^ year	440	342	337	98.5
5^th^ year	420	345	341	98.8
Total	2400	1810	1700	93.9

**Table 4:** Knowledge and perception about dental implants among undergraduate students from 1^st^ year to 5^th^ year

Questions	*N* (%)	1^st^ year *n* (%)	2^nd^ year *n* (%)	3^rd^ year *n* (%)	4^th^ year *n* (%)	5^th^ year *n* (%)
*How well informed are you about dental implants?*						
1. Very well	80 (4.7)	4 (1.1)	11 (3.5)	11 (3.3)	18 (5.3)	36 (10.6)
2. Well	283 (16.6)	26 (6.9)	43 (13.6)	57 (17.3)	8374 (22.0)	83 (24.3)
3. Moderately Well	928 (54.6)	161 (42.8)	158 (50.0)	189 (57.3)	218 (64.7)	202 (59.2)
4. Poorly	358 (21.1)	150 (39.9)	95 (30.1)	68 (20.6)	26 (7.7)	19 (5.6)
5. Not at all	51 (3.0)	35 (9.3)	9 (2.8)	5 (1.5)	1 (0.3)	1 (0.3)
*What do you think is the main advantage of dental implants as compared to other tooth-replacement modalities?*						
1. Aesthetics; looks nicer	245 (14.4)	115 (30.6)	38 (12.0)	32 (9.7)	34 (10.1)	26 (7.6)
2. More conservative	469 (27.6)	82 (21.8)	77 (24.4)	93 (28.2)	73 (21.7)	144 (42.2)
3. Longevity; lasts longer	902 (53.1)	137 (36.4)	183 (57.9)	194 (58.8)	223 (66.2)	165 (48.4)
4. Do not know	84 (5.0)	42 (11.2)	18 (5.7)	11 (3.3)	7 (2.1)	6 (1.7)
*What do you think is the most important factor for implant success?*						
1. Case selection	427 (25.1)	55 (14.6)	31 (9.8)	41 (12.4)	96 (28.5)	204 (59.8)
2. Implant type and material	543 (31.9)	142 (37.8)	126 (39.9)	160 (48.5)	66 (19.6)	49 (14.4)
3. Patient compliance	211 (12.4)	42 (11.2)	44 (13.9)	45 (13.6)	46 (13.6)	34 (10.0)
4. Surgical technique	182 (10.7)	71 (18.9)	51 (16.1)	23 (7.0)	20 (5.9)	17 (5.0)
5. Experience of operator	228 (13.4)	31 (8.2)	29 (9.2)	33 (10.0)	101 (30.0)	34 (10.0)
6. Do not know	109 (6.4)	35 (9.3)	35 (11.1)	28 (8.5)	8 (2.4)	3 (0.9)
*What do you tell your patient is the longevity of dental implants?*						
1. 2–5 yrs	59 (3.5)	32 (8.5)	3 (0.9)	17 (5.2)	0 (0.0)	7 (2.1)
2. 5–10 yrs	349 (20.5)	94 (25.0)	72 (22.8)	81 (24.5)	37 (11.0)	65 (19.1)
3. 10–20 yrs	586 (34.5)	76 (20.2)	95 (30.1)	98 (29.7)	153 (45.4)	164 (48.1)
4. Life-time	429 (25.2)	105 (27.9)	83 (26.3)	62 (18.8)	95 (28.2)	84 (24.6)
5. Do not know	277 (16.3)	69 (18.4)	63 (19.9)	72 (21.8)	52 (15.4)	21 (6.2)
*Do you feel that dental implants require additional oral hygiene maintenance and care by the patient and dentist?*						
1. No, are cleaned like natural teeth	413 (24.3)	84 (22.3)	89 (28.2)	89 (27.0)	85 (25.2)	66 (19.4)
2. Yes, need more care than natural teeth	993 (58.4)	243 (64.6)	137 (43.4)	196 (59.4)	161 (47.8)	256 (75.1)
3. No, needless care than natural teeth	101 (5.9)	3 (0.8)	33 (10.4)	16 (4.8)	44 (13.1)	5 (1.5)
4. Do not know	193 (11.4)	46 (12.2)	57 (18.0)	29 (8.8)	47 (13.9)	14 (4.1)

**Table 5:** Knowledge and perception about dental implants among undergraduate students from 1^st^ year to 5^th^ year

Questions	*N* (%)	1^st^ year *n* (%)	2^nd^ year *n* (%)	3^rd^ year *n* (%)	4^th^ year *n* (%)	5^th^ year *n* (%)
*On a scale of 1–10, how difficult do you feel is it to place implants as compared to other dental procedures?*						
1. 1 = very easy	39 (2.3)	6 (1.7)	8 (2.5)	2 (0.6)	4 (1.2)	19 (5.6)
2. 5 = average	966 (56.8)	198 (52.6)	155 (49.1)	165 (50.0)	250 (74.2)	198 (58.1)
3. 10 = very difficult	695 (40.9)	172 (45.7)	153 (48.4)	163 (49.4)	83 (24.6)	124 (36.4)
*What is the cost of procuring a dental implant from an implant company?*						
1. Rs. 6000–10,000 ($60–100)	107 (6.3)	28 (7.4)	18 (5.7)	29 (8.8)	15 (4.5)	17 (5.0)
2. Rs. 10,000–15,000 ($100–150)	176 (10.4)	27 (7.2)	27 (8.5)	30 (9.1)	33 (9.8)	59 (17.3)
3. Rs. 15,000–20,000 ($150–200)	268 (15.8)	80 (21.3)	28 (8.9)	43 (13.0)	51 (15.1)	66 (19.4)
4. Rs. 20,000–25,000 ($200–250)	552 (32.5)	85 (22.6)	111 (35.1)	90 (27.3)	132 (39.2)	134 (39.3)
5. Do not know	597 (35.1)	156 (41.5)	132 (41.8)	138 (41.8)	106 (31.5)	65 (19.1)
*How much do you feel is the initial set-up cost required to incorporate implant surgery into practice?*						
1. Rs. 200,000–300,000 ($2000–3000)	554 (32.6)	96 (25.5)	91 (28.8)	170 (51.5)	97 (28.8)	100 (29.3)
2. Rs. 400,000–500,000 ($4000–5000)	319 (18.8)	61 (16.2)	66 (20.9)	45 (13.6)	78 (23.1)	69 (20.2)
3. Rs. 500,000–1,000,000 ($5000–10,000)	496 (29.2)	109 (29.0)	88 (27.8)	66 (20.0)	108 (32.0)	125 (36.7)
4. Rs. 1,000,000 and above (≥$10,000)	331 (19.5)	110 (29.3)	71 (22.5)	49 (14.8)	54 (16.0)	47 (13.8)
*Do you think that dental implants are an acceptable solution for missing teeth in the Nepalese scenario?*						
1. Yes, implants are here to stay	422 (24.8)	124 (33.0)	97 (30.7)	81 (24.5)	50 (14.8)	70 (20.5)
2. No, economic feasibility will limit their usage	1148 (67.5)	219 (58.2)	204 (64.6)	226 (68.5)	252 (74.8)	247 (72.4)
3. No, too invasive for patient acceptance	114 (6.7)	31 (8.2)	14 (4.4)	21 (6.4)	31 (9.2)	17 (5.0)
4. No, other reasons: (please specify)	16 (0.9)	2 (0.5)	1 (0.3)	2 (0.6)	4 (1.2)	7 (2.1)

A majority of the total students perceived themselves to be moderately well informed about dental implants (54.6%). A majority thought the main advantage of dental implants as compared to other tooth-replacement modalities is longevity (53.1%), only 27.6% said more conservative than other tooth-replacement modalities. A majority (58.4%) felt that dental implants require additional oral hygiene maintenance and care by the patient and dentist than natural teeth. 56.8% perceived the difficulty encountered to place implants as compared to other dental procedures to be average. Over two thirds (67.5%) thought that economic feasibility will limit use of dental implants in Nepal. There were differences in the perception and knowledge at different academic levels, but not as expected (Tables 4, 5).

## Discussion

This study shows that there was a predominance of females and higher number of students at 5^th^ year and 4^th^ year present than at initial years (out of total students at that year). Differences were seen in the responses of students at different academic levels but, not as expected. It was expected to get a higher number of most appropriate evidence-based answers from the students at higher academic years.

A majority of all students and 48.4% of 5^th^ year said longevity is the main advantage of dental implants. Of the total students, 25.2% and 24.6% of the 5^th^ year said implants lasts a life-time. Literature shows that the main advantage of dental implants as compared to other tooth-replacement modalities is they are more conservative as there is no need of preparing natural teeth as in conventional partial dentures.^1,15,17^ Duration of longitudinal studies on survival of implants in the literature is upto 20 years.^18–20^ So, the expected answer for the longevity of dental implants was 10–20 years. Patients should not be told that a dental implant will last for a life-time. Such belief will lead to unnatural patients’ expectations.

Similarly, evidence shows that the most important factor for implant success is case selection,^2,21,22^ but the highest percentage of the total students (31.9%) said ‘implant type and material,’ and 40.2% of students at 5^th^ year gave other answers than case selection. To the question about the cost of procuring a dental implant from an implant company and the initial set-up cost required to incorporate implant surgery into practice, a majority was not gained by any response. The highest percentage of the total respondents (35.1%) said they do not know the cost of procuring a dental implant. Such responses show their poor clinical exposure related to dental implants and a need to expose them to dental implant cases.

A majority of the total students and 72.4% of 5^th^ year said they do not think dental implants are an acceptable solution for missing teeth in the Nepalese scenario because economic feasibility will limit their usage. A systematic review of literature has shown general public concern about the high cost of dental implant therapy,^23^ but dental students should must be taught about the long-term cost of other treatment modalities as compared to implants so that they can advise patients about implant therapy whenever appropriate.

In a similar study done to assess the knowledge of dental interns of Nepal 58.6% said the main advantage of dental implants as compared to other tooth-replacement modalities is they are more conservative and 51.07% said case selection to be the most important factor for implant success.^24^ This suggests that undergraduate dental students in Nepal acquire much of their basic knowledge about dental implants during their internship program. They have perceived the need and shown a positive attitude towards gaining more information about dental implant procedures through various sources.^16^ An all-India survey carried out to gauge the knowledge and perception of undergraduate students towards dental implants also concluded that there is a need for revision of undergraduate curriculum.^15^

A survey of American dental schools conducted to determine the curricular structure, teaching philosophies, and materials used in undergraduate implant dentistry courses showed that in 70% of the dental schools this course was offered in the third year of the undergraduate dental curriculum and for 75% of the schools, the duration of the course ranged from 3 to 6 months.^10^ In 78% of the schools, a laboratory course was offered in conjunction with the implant course and the majority of schools (88%) allowed undergraduate students to restore implant cases clinically; single-tooth implant restoration being the most popular type of implant restoration for 78% of the schools.^10^

Another survey of European dental schools conducted to determine the curricular structure, teaching philosophies, and materials used in undergraduate implant dentistry courses found that undergraduate implant dentistry educational programs varied from school to school, yet a large percentage of schools agreed on certain topics, including the importance of including implant education in undergraduate dental programs.^11^ The First European Workshop on Implant Dentistry University Education held in Prague on 19–22 June 2008 released consensus document recommending that implant dentistry should be an integral part of the undergraduate curriculum.^12^

The Australian Consensus Workshop on Implant Dentistry University Education, Gold Coast, 4–6 February 2010 also released consensus document recommending key competencies (knowledge, skills, attitudes and values) in the field of implant dentistry, necessary for graduating general practitioners in Australia.^13^

Not only developed countries but also developing countries need to follow clear recommendations and guidelines for implementation of implant dentistry in undergraduate curriculum. Though many obstacles like inadequate curriculum time, lack of financial resources, lack of qualified faculty are there making the job really challenging in developing countries.^14^ It is necessary to come-up with solutions or alternatives to those obstacles as soon as possible.

## Conclusions

Knowledge and perception about dental implants among undergraduate dental students differed at different academic years, but not as expected. Knowledge about dental implants was expected to increase with increase in undergraduate training but this was not observed. Even at the late-clinical year a majority of students gave unsatisfactory answers. Thus, there is a need for curriculum review, evaluation of teaching materials and methods, consensus workshops drawing solutions to obstacles and providing recommendations and clear guidelines to include implant dentistry in undergraduate curriculum of developing countries like Nepal, so that students will be able to respond properly to the increasing number of patients with queries about dental implants.

## Supplementary information

NIH-Certificate

## Data Availability

Data will be made available on request.
