# Supplementary figures and images for: Knowledge and perception about dental implants among undergraduate dental students
Source: BDJ Open. 2019 Mar 14;5:1. doi: 10.1038/s41405-018-0009-1 (PMC6418164; doi:10.1038/s41405-018-0009-1)

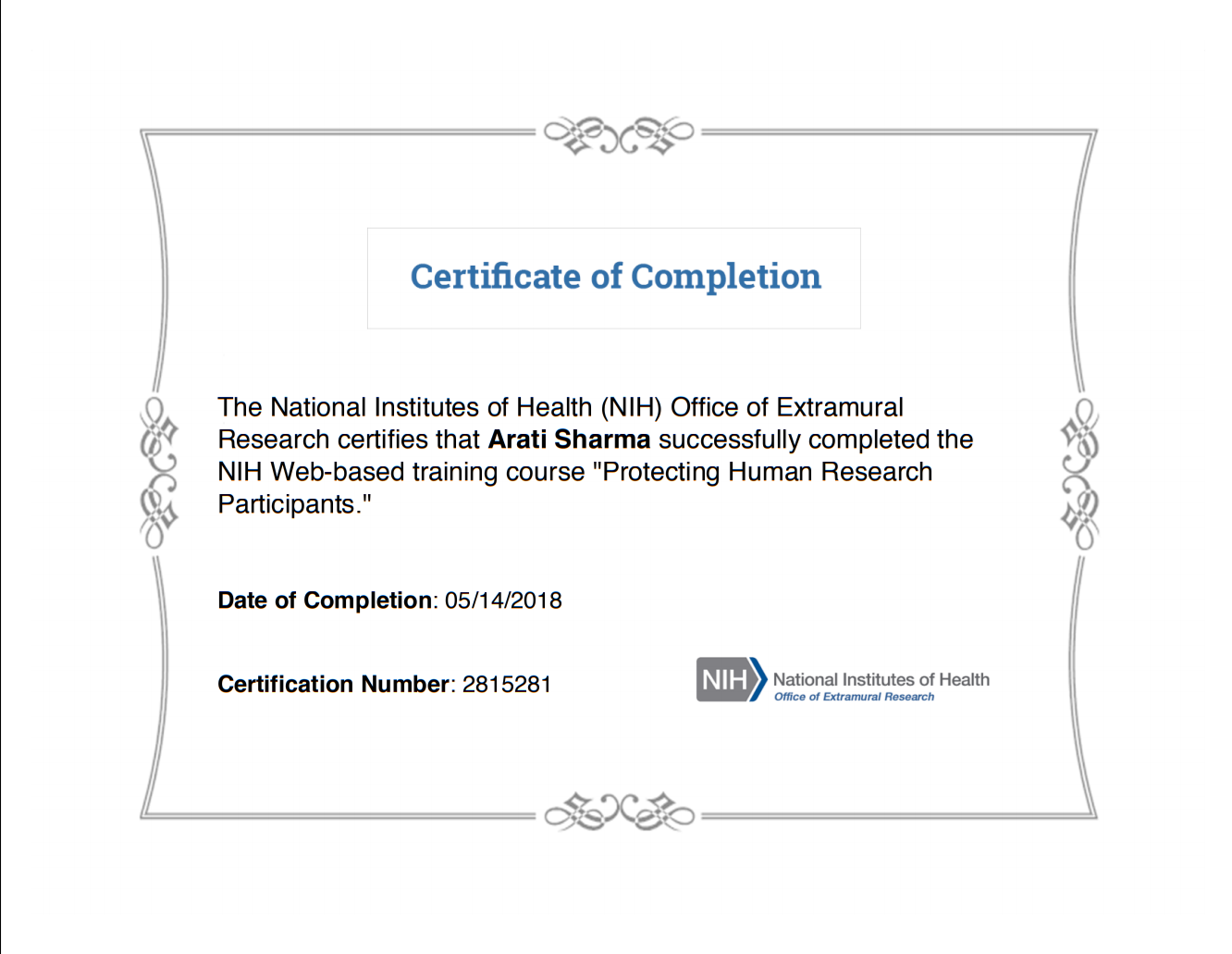

Supplement: Supplementary file 1 — NIH-Certificate [file 41405_2018_9_MOESM1_ESM.png]
